# Complete Genome Sequence of the Nosocomial Pathogen Acinetobacter nosocomialis Strain M2

**DOI:** 10.1128/MRA.00538-19

**Published:** 2019-10-31

**Authors:** Bailey Pehde, Nicholas Lizer, Michael Carruthers

**Affiliations:** a Department of Microbiology and Immunology, College of Osteopathic Medicine, Des Moines University, Des Moines, Iowa, USA; University of Rochester School of Medicine and Dentistry

## Abstract

Acinetobacter nosocomialis is an opportunistic human pathogen that is part of the Acinetobacter calcoaceticus/Acinetobacter baumannii (ACB) complex. Here, we report the complete genome sequence of Acinetobacter nosocomialis strain M2.

## ANNOUNCEMENT

Acinetobacter nosocomialis is a Gram-negative coccobacillus that is a member of the Acinetobacter calcoaceticus/Acinetobacter baumannii (ACB) complex. While A. baumannii predominates over all other members of the ACB complex in terms of incidence, poorer clinical outcomes, and antibiotic resistance rates, A. nosocomialis remains a clinically relevant human pathogen.

Previously, we reported the draft genome sequence of A. nosocomialis strain M2 (1). Here, we report the complete genome sequence of A. nosocomialis strain M2. Strain M2 is one of the most well-characterized A. nosocomialis strains to date, with published research detailing quorum sensing (2), type IV pili (3, 4), type VI secretion (5), antibiotic resistance (6), oxidative stress (7), surface motility (8), type II secretion (9, 10), type I secretion, and contact-dependent inhibition systems (11).

An ice scraping from a frozen stock of strain M2 stored at −80°C was struck for isolation onto an LB agar (Miller) plate and incubated at 37°C for ∼16 h. A 14-ml round-bottom tube containing 2 ml of Luria broth was inoculated with a single colony picked from this plate and incubated at 37°C and 180 rpm for ∼16 h. This overnight culture was used to inoculate a 250-ml Erlenmeyer flask containing 25 ml of Luria broth prewarmed to 37°C to a starting optical density at 600 nm (OD_600_) of 0.05, as measured by a BioMate 3 spectrophotometer (Thermo Fisher Scientific). This was incubated at 37°C and 180 rpm until an OD_600_ of 0.7 was reached. DNA extraction was performed using 5 ml of this culture and the Genomic-tip 500 kit (Qiagen) per the manufacturer’s instructions. Purified genomic DNA (gDNA) was analyzed using a NanoDrop spectrophotometer and Qubit fluorometer (Thermo Scientific), the latter using a double-stranded DNA (dsDNA) high-sensitivity (HS) kit per the manufacturer’s instructions. A 1D native gDNA barcoded library was generated using an Oxford Nanopore Technologies (ONT) native barcoding expansion kit (catalog number EXP-NBD104) and ligation sequencing kit (catalog number SQK-LSK109) per the manufacturer’s instructions. The DNA library containing the NB01 barcode was sequenced on a FLO-MIN106 flow cell using the MinION device (ONT). This sequencing run was multiplexed with 1 other bacterial gDNA library tagged with a different barcode. All computation, other than data collection, was performed using Amazon’s Elastic Compute Cloud (EC2) service. Base calling was performed using Guppy v3.0.3 (ONT) with default settings and graphics processing unit (GPU) acceleration (EC2 p3.2xlarge instance). Demultiplexing was performed first using Guppy, which yielded 176,357 reads totaling ∼2.9 Gbp. An additional round of demultiplexing, with adapter and barcode trimming, was performed with Porechop v0.2.4 (github.com/rrwick/Porechop) with the discard_middle option enabled. Reads were filtered using Filtlong v0.2.0 (github.com/rrwick/Filtlong) with standard settings, except for a minimum length of 20,000 bp, to keep only the top 90% of reads, and with a target output of 500 Mbp. Canu v1.8 (12), with standard settings and an estimated genome size of 3.8 Mbp, was used to correct and trim reads. Reads were then assembled using Unicycler v0.4.7 (13) with standard settings for long reads, including error correction. Polishing of the assembly was carried out using medaka v0.6.5 (ONT), followed by Pilon v1.23 (14) with standard settings, the latter using Illumina MiSeq reads that were originally used to create the previously described strain M2 draft genome assembly (1). All trimmed reads from both sequencing runs were mapped onto the final assembly using Minimap2 v2.1.7 (15) and Bowtie2 v2.3.5.1 (16), with standard settings, for ONT MinION reads and Illumina MiSeq reads, respectively. Average coverage depth of the A. nosocomialis genome, as calculated by SAMtools v1.9 (17), was 720× with ONT reads and 331× with Illumina reads. The final assembly was annotated using the NCBI Prokaryotic Genome Annotation Pipeline v4.8 (18, 19). The complete assembled genome of A. nosocomialis strain M2 consists of one circular chromosome of 3,824,973 bp having a 38.8% GC content and 3,458 protein-encoding genes.

### Data availability.

The complete genome sequence of A. nosocomialis strain M2 reported here was deposited in GenBank under accession number CP040105. Base-called reads can be found in the Sequence Read Archive under accession number SRR9018422.
